# Report from Nairobi: towards a 25% reduction in uncontrolled hypertension in Africa

**Published:** 2018

**Authors:** Marí Hudson

‘The first step in an ambitious, long-term, intercontinental project to save millions of lives in Africa by reducing the unacceptably high stroke morbidity and mortality rates and other devastating but preventable consequences of undiagnosed and uncontrolled hypertension in Africa. Indeed an important step towards the ultimate goal: a 25% reduction in cases of uncontrolled hypertension on this continent by 2025, as set out in the PASCAR Roadmap on Hypertension.’^1^

This is how the outcome can be described of the first continental meeting in Nairobi, where a delegation of more than 30 key opinion leaders in hypertension and cardiology from Britain, Ireland, India and Africa deliberated for two days to review and Africanise the successful and relevant Indian Certificate Course in the Management of Hypertension (ICCMH*). This emerged during a media conference held at the meeting.

Representatives of the Public Health Foundation of India (PHFI) in New Delhi, the International Society of Hypertension (ISH), the British and Irish Hypertension Society (BIHS), the Centre for Chronic Disease Control (CCDC) in New Delhi and the Pan-African Society of Cardiology (PASCAR) gathered in Nairobi to discuss the expansion and customisation of the ICCMH to the African region.

The main objective of the course is to train doctors, nurses and community healthcare workers on the continent to diagnose and manage hypertension as effectively as possible at the primary care level, by early diagnosis and better hypertension control, based on guidelines adapted for the African continent and different ethnic groups. This will free the few hypertension specialists on the continent to manage complicated hypertension cases at a tertiary level. The following key aspects were highlighted at the media conference.

## Undiagnosed and uncontrolled hypertension is the number one killer in the world 

In excess of 10 million people around the world die each year of hypertension-related diseases, mainly cardiovascular diseases, including stroke. It kills more people in the world than any other communicable disease, said Prof Francesco P Cappuccio, president of the BIHS.

## The World Health Organisation has declared Africa the region of the world with the highest incidence of hypertension

The African Union considers hypertension the most important challenge on the continent after HIV/AIDS, but up to now, experts have not succeeded in translating these political decisions into important actions that could change cardiovascular health on the continent, explained Prof Saad Subahi, cardiologist and president of PASCAR’s National Council. This is where PASCAR entered four years ago and initiated the PASCAR Roadmap on Hypertension.

A recent situation analysis revealed that hypertension affects one-third (150 million) of the adult population in Africa, but only one-tenth (about 15 million) of these people are aware of the disease, and when they are aware, only one in five people (about three million) is treated. When they are treated, only one in 14 (about 215 000) is treated to such an extent that their risk for renal disease, heart disease or stroke, or dying from hypertension are reduced. This is a dire situation, Prof Subahi said.

## Increased awareness among health professionals and society was identified as the highest priority towards a 25% reduction in uncontrolled hypertension by 2025 

In the first attempt to increase awareness, more than 20 African countries took part in a huge global initiative in 2017 to screen people for hypertension. This will now be followed by the Africanisation, translation and roll out of the Indian hypertension-education programme in Africa over the next two years, said Prof Neil Poulter, president of the ISH.

## International hypertension experts from the ISH, BHIS and India are keen and commited to assist Africa in its fight against hypertension 

‘It is our duty to fill a gap. A 25% reduction in undiagnosed, uncontrolled hypertension will mean saving the lives of millions of people every year. It will also reduce morbidity by millions,’ Cappucino reiterated.

## The next steps

Following the meeting in Nairobi, the priority actions, as identified in the PASCAR Roadmap on Hypertension, will be implemented to overcome the identified roadblocks. These key elements were highlighted at the media conference:

- Training and education to increase awareness: PASCAR will roll out the adapted training programme to train the trainers across the African region in the coming months; to ultimately train 250 000 community health workers, 50 000 nurses and 25 000 certified general physicians.
- Create and customise easy-to-use treatment protocols for different ethnic groups within a proper referral linkage pattern. Poulter shed more light on the possible treatment protocols: ‘The CREOLE trial^2^ in six sub-Saharan African countries, to indicate which antihypertensive drugs and combination drugs will be best for African patients and even for different ethnic groups, will be completed in June 2018. The results, as well as the availability and cost effectiveness of drugs, will be taken into account when drafting the treatment protocols.’
  ‘We expect the CREOLE trial to show that a diuretic and a calcium channel blocker (CCB) will be the most effective anti-hypertensive drugs for black patients, but we don’t know yet what combination will be best: a CCB and a diuretic, or a CCB plus angiotensin converting enzyme inhibitor (ACEI), or a diuretic plus an ACEI. If we get this information, it will be a big leap forward. We would like to reproduce the CREOLE trial in South Asia and the Far East, to determine which two drugs are the best for each ethnic group.’
- High-quality generic anti-hypertensive drugs will be produced and delivered cost effectively by non-profit-making companies. These efforts will hopefully lead to more countries in Africa joining the initiative. Currently only 25% of countries in Africa have a government policy and guidelines to treat and reduce the burden of hypertension,’ Poulter said.
